# Multiomics Characterization of Preterm Birth in Low- and Middle-Income Countries

**DOI:** 10.1001/jamanetworkopen.2020.29655

**Published:** 2020-12-18

**Authors:** Fyezah Jehan, Sunil Sazawal, Abdullah H. Baqui, Muhammad Imran Nisar, Usha Dhingra, Rasheda Khanam, Muhammad Ilyas, Arup Dutta, Dipak K. Mitra, Usma Mehmood, Saikat Deb, Arif Mahmud, Aneeta Hotwani, Said Mohammed Ali, Sayedur Rahman, Ambreen Nizar, Shaali Makame Ame, Mamun Ibne Moin, Sajid Muhammad, Aishwarya Chauhan, Nazma Begum, Waqasuddin Khan, Sayan Das, Salahuddin Ahmed, Tarik Hasan, Javairia Khalid, Syed Jafar Raza Rizvi, Mohammed Hamad Juma, Nabidul Haque Chowdhury, Furqan Kabir, Fahad Aftab, Abdul Quaiyum, Alexander Manu, Sachiyo Yoshida, Rajiv Bahl, Anisur Rahman, Jesmin Pervin, Jennifer Winston, Patrick Musonda, Jeffrey S. A. Stringer, James A. Litch, Mohammad Sajjad Ghaemi, Mira N. Moufarrej, Kévin Contrepois, Songjie Chen, Ina A. Stelzer, Natalie Stanley, Alan L. Chang, Ghaith Bany Hammad, Ronald J. Wong, Candace Liu, Cecele C. Quaintance, Anthony Culos, Camilo Espinosa, Maria Xenochristou, Martin Becker, Ramin Fallahzadeh, Edward Ganio, Amy S. Tsai, Dyani Gaudilliere, Eileen S. Tsai, Xiaoyuan Han, Kazuo Ando, Martha Tingle, Ivana Marić, Paul H. Wise, Virginia D. Winn, Maurice L. Druzin, Ronald S. Gibbs, Gary L. Darmstadt, Jeffrey C. Murray, Gary M. Shaw, David K. Stevenson, Michael P. Snyder, Stephen R. Quake, Martin S. Angst, Brice Gaudilliere, Nima Aghaeepour

**Affiliations:** 1 Department of Pediatrics and Child Health, Aga Khan University, Karachi, Pakistan; 2 Centre for Public Health Kinetics, New Delhi, Delhi, India; 3 International Center for Maternal and Newborn Health, Department of International Health, Johns Hopkins Bloomberg School of Public Health, Baltimore, Maryland; 4 Public Health Laboratory-Ivo de Carneri, Pemba Island, Zanzibar; 5 Maternal, Newborn, Child and Adolescent Health Research, World Health Organization, Geneva, Switzerland; 6 Matlab Health Research Centre, International Centre for Diarrhoeal Disease Research, Dhaka, Bangladesh; 7 Maternal and Child Health Division, International Centre for Diarrhoeal Disease Research, Dhaka, Bangladesh; 8 Department of Obstetrics and Gynecology, University of North Carolina at Chapel Hill, Chapel Hill; 9 School of Public Health, University of Zambia, Lusaka, Zambia; 10 Global Alliance to Prevent Prematurity and Stillbirth, Seattle, Washington; 11 Department of Anesthesiology, Perioperative and Pain Medicine, Stanford University School of Medicine, Stanford, California; 12 Digital Technologies Research Centre, National Research Council Canada, Toronto, Ontario, Canada; 13 Department of Bioengineering, Stanford University, Stanford, California; 14 Department of Genetics, Stanford University School of Medicine, Stanford, California; 15 Division of Neonatal and Developmental Medicine, Department of Pediatrics, Stanford University School of Medicine, Stanford, California; 16 Stanford University School of Medicine, Stanford, California; 17 Department of Obstetrics and Gynecology, Stanford University School of Medicine, Stanford, California; 18 Department of Pediatrics, University of Iowa, Iowa City; 19 Department of Biomedical Informatics, Stanford University School of Medicine, Stanford, California

## Abstract

**Importance:**

Worldwide, preterm birth (PTB) is the single largest cause of deaths in the perinatal and neonatal period and is associated with increased morbidity in young children. The cause of PTB is multifactorial, and the development of generalizable biological models may enable early detection and guide therapeutic studies.

**Objective:**

To investigate the ability of transcriptomics and proteomics profiling of plasma and metabolomics analysis of urine to identify early biological measurements associated with PTB.

**Design, Setting, and Participants:**

This diagnostic/prognostic study analyzed plasma and urine samples collected from May 2014 to June 2017 from pregnant women in 5 biorepository cohorts in low- and middle-income countries (LMICs; ie, Matlab, Bangladesh; Lusaka, Zambia; Sylhet, Bangladesh; Karachi, Pakistan; and Pemba, Tanzania). These cohorts were established to study maternal and fetal outcomes and were supported by the Alliance for Maternal and Newborn Health Improvement and the Global Alliance to Prevent Prematurity and Stillbirth biorepositories. Data were analyzed from December 2018 to July 2019.

**Exposures:**

Blood and urine specimens that were collected early during pregnancy (median sampling time of 13.6 weeks of gestation, according to ultrasonography) were processed, stored, and shipped to the laboratories under uniform protocols. Plasma samples were assayed for targeted measurement of proteins and untargeted cell-free ribonucleic acid profiling; urine samples were assayed for metabolites.

**Main Outcomes and Measures:**

The PTB phenotype was defined as the delivery of a live infant before completing 37 weeks of gestation.

**Results:**

Of the 81 pregnant women included in this study, 39 had PTBs (48.1%) and 42 had term pregnancies (51.9%) (mean [SD] age of 24.8 [5.3] years). Univariate analysis demonstrated functional biological differences across the 5 cohorts. A cohort-adjusted machine learning algorithm was applied to each biological data set, and then a higher-level machine learning modeling combined the results into a final integrative model. The integrated model was more accurate, with an area under the receiver operating characteristic curve (AUROC) of 0.83 (95% CI, 0.72-0.91) compared with the models derived for each independent biological modality (transcriptomics AUROC, 0.73 [95% CI, 0.61-0.83]; metabolomics AUROC, 0.59 [95% CI, 0.47-0.72]; and proteomics AUROC, 0.75 [95% CI, 0.64-0.85]). Primary features associated with PTB included an inflammatory module as well as a metabolomic module measured in urine associated with the glutamine and glutamate metabolism and valine, leucine, and isoleucine biosynthesis pathways.

**Conclusions and Relevance:**

This study found that, in LMICs and high PTB settings, major biological adaptations during term pregnancy follow a generalizable model and the predictive accuracy for PTB was augmented by combining various omics data sets, suggesting that PTB is a condition that manifests within multiple biological systems. These data sets, with machine learning partnerships, may be a key step in developing valuable predictive tests and intervention candidates for preventing PTB.

## Key Points

**Question:**

What maternal biological modalities are associated with preterm birth (PTB)?

**Findings:**

In this diagnostic/prognostic study of 81 pregnant women from 5 birth cohorts in low- and middle-income countries, several correlates of preterm birth in urine and blood were found to be associated with PTB. Although cohort-specific signatures were present, a machine learning algorithm was able to generate a model that was capable of predicting PTB across the cohorts.

**Meaning:**

Results of this study suggest that most PTBs can be predicted using blood and urine samples collected early in the pregnancy, providing opportunities for interventions.

## Teaser

This diagnostic/prognostic study describes the use of cell-free transcriptomics, urine metabolomics, and plasma proteomics for identifying the biological measurements associated with preterm birth.

## Introduction

Preterm birth (PTB) is defined by the World Health Organization as the delivery of a live infant before the completion of 37 weeks of gestation.^1,2^ The worldwide rate of PTB in 2014 was estimated to be 10.6% (uncertainty interval, 9.0%-12.0%), with 80% of all cases occurring in South Asia and sub-Saharan Africa.^2^ Many risk factors for PTB have been highlighted in previous studies and include obstetrical (eg, previous PTB and multiple gestation), medical (eg, maternal obesity, diabetes, and chronodisruption), and external (eg, smoking and maternal stress) conditions.^3,4,5,6,7,8,9^ For example, a meta-analysis of individual- and population-level attributes among 4.1 million births concluded that “unknown factors requiring further research to act upon account for ~2/3 of the preterm birth rate.”^10^^(p13)^ Unveiling and elucidating the role of early biological antecedents of PTB has been deemed a necessary step toward developing new diagnostic tests and therapeutic interventions.^11,12,13^ Biological investigations into the mechanisms of PTB are complicated, as indicated by accumulating evidence that distinct patient subpopulations follow divergent biological trajectories.^14,15^ Given this heterogeneity, simultaneously studying diverse cohorts is critical for identification of generalizable biological pathways.^16^

Recent technological advances have enabled the characterization of a broad range of biological changes during pregnancy. Biological layers explored include single-cell profiling of signaling pathways,^17^ measurements of plasma cell-free ribonucleic acid (cfRNA),^18^ proteome^19,20^ and metabolome^21^ characterization of the microbiome,^14,22^ and detailed genomics analysis.^23^ In addition, a recent multiomics investigation demonstrated that biological changes during normal pregnancy involve a number of intricate interactions of biological processes, which can be measured using a coordinated set of assays.^24^ The integration of the large, multidimensional data sets generated in a multiomics setting requires complex machine learning pipelines that will remain robust in the face of the inconsistent intrinsic properties of these high-throughput assays and cohort-specific variations.^15^

To our knowledge, this is the first multiomics analysis of term and preterm pregnancies from multiple cohorts in low- and middle-income countries (LMICs). These cohorts were established using biorepositories of samples and phenotypic data for studying maternal and fetal outcomes collected and stored from diverse populations of South Asia and sub-Saharan Africa. The study aimed to investigate the ability of transcriptomics and proteomics profiling of blood plasma and metabolomics analysis of urine to identify early biological measurements associated with PTB.

## Methods

Approval was obtained from the Stanford University Institutional Review Board, and ethical exemptions were sought and obtained independently from the respective country by each birth cohort supported by the Alliance for Maternal and Newborn Health Improvement (AMANHI) and the Global Alliance to Prevent Prematurity and Stillbirth (GAPPS) biorepositories. Written informed patient consent was obtained from each participant in the original cohorts and extends to the present study. We followed the Transparent Reporting of a Multivariable Prediction Model for Individual Prognosis or Diagnosis (TRIPOD) reporting guideline. This study analyzed plasma and urine samples collected from May 2014 to June 2017, and data were analyzed from December 2018 to July 2019.

### Participants and Study Design

The study population comprised pregnant women selected from 5 biorepository-supported cohorts in Matlab, Bangladesh; Lusaka, Zambia; Sylhet, Bangladesh; Karachi, Pakistan; and Pemba, Tanzania. No compensation or incentives were provided for participating in this study.

**Figure 1.**
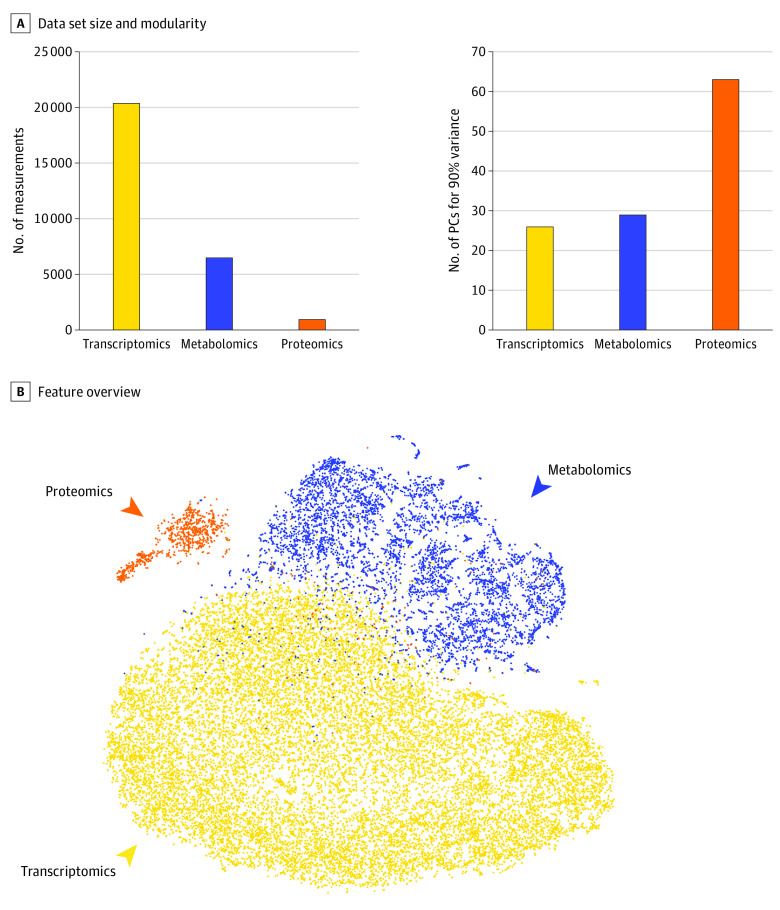
Study Overview A, The 3 data sets (plasma cell-free ribonucleic acid [cfRNA] or transcriptomics, metabolomics, and proteomics) produced a number of different features and had a range of correlations among the measured features. The internal correlation between features from each data set was quantified using the number of principal components (PCs) needed to capture 90% variance (eg, the cf-RNA data set had the most features but was highly correlated internally; therefore, fewer PCs were needed). B, A 2-dimensional representation of all measurements demonstrates the correlation between subsets of urine metabolites and cfRNA detected in plasma as well as a limited number of plasma proteins.

Plasma samples were assayed to measure targeted proteins and cfRNA, and urine samples were analyzed for metabolites. The cfRNA analysis resulted in 20 659 measurements, the targeted proteomics assay measured 1002 proteins in plasma, and 6630 metabolites were measured in urine. The number of measurements of these assays did not correlate with their modularity, as indicated by the number of principal components needed to account for 90% of the total variance (Figure 1A). This result highlighted the need for a 2-layer metadimensional integrative approach to prevent the assays with more measurements to bias the predictive models (eMethods in the Supplement). An overview of the entire data set was produced by first calculating a correlation network of all available measurements and then producing a 2-dimensional layout for visualization using the t-SNE^25^ algorithm (Figure 1B).

### Biological Assays

From all AMANHI and GAPPS cohorts, trained phlebotomists collected blood samples for centrifugation and aliquoting of serum, plasma, and buffy coat for storage and future analyses. In addition, maternal urine was collected in parallel. Collection and processing of all sample types were performed according to harmonized operating procedures at all study cohorts. The eMethods in the Supplement provides details on the biological assays.

### Statistical Analysis 

Data were analyzed from December 2018 to July 2019. All analyses were performed with R, version 3.6.1 (R Foundation for Statistical Computing). All multivariate modeling was performed with a 2-layer cross-validation strategy to prevent overfitting of the data and to ensure generalizability. Mixed-effect models were used to account for cohort-specific variations (eMethods in the Supplement). The analysis is independently reproducible. The measured features from all 3 omics data sets (transcriptomics, metabolomics, and proteomics); the algorithms and source codes for reproduction of the results; and an interactive website for visualizing the entire data set, the feature evaluation scores for PTB and gestational age (GA) at sampling, and the pathway enrichment analysis are available online (https://nalab.stanford.edu/multiomicsmulticohortpreterm/).

We used linear discriminant analysis and principal component analysis (PCA), respectively, to create a 2-dimensional representation of the entire cohort with cohort labels as the supervised guide and without supervised information. To confirm the presence of cohort-specific signatures, we used random forest analysis. We created models for each patient to estimate GA at the time of sample collection. To simultaneously optimize the integrative model and test the performance of the model on previously unseen patients, we applied a cross-validation strategy. To predict PTB (GA at delivery <37 weeks), we used a leave-one-out cross-validation procedure to test the models on blinded participants.

## Results

Of the 81 pregnant women included in this study, 39 had PTBs (48.1%) and 42 had term pregnancies (51.9%). The mean (SD) maternal age was 24.8 (5.3) years. The median sampling time was 13.6 weeks of gestation, according to ultrasonography (Figure 1A).

### Data Quality Assessment

To investigate cohort-specific data signatures, PCA was used to create a 2-dimensional representation of the entire cohort for each biological modality and all modalities combined (eFigure 1A in the Supplement). The PCA demonstrated that the largest source of variation in the data was not driven by fundamental differences between the cohorts. Supervised linear discriminant analysis^26^ confirmed the existence of more subtle cohort-specific signatures that were not statistically significant enough to be visualized in an unsupervised PCA (eFigure 1B in the Supplement). The presence of cohort-specific signatures was confirmed using random forest analysis^27^ that underwent cross-validation to predict the cohort from which the patient was selected exclusively on the basis of each biological modality (eFigure 1C in the Supplement).

The impact of sample storage time was quantified with random forest analysis that underwent cross-validation in which the number of days between sample collection and laboratory analyses was used as a continuous prediction target. The results were statistically significant (thresholds of *P* = 1.25762 × 10^−01^ for transcriptomics, *P* = 8.83433 × 10^−06^ for metabolomics, and *P* = 5.56758 × 10^−02^ for proteomics) only in the case of the urine metabolomics data set, indicating the potential for sample degradation over time (eFigure 1D in the Supplement). However, this result did not confound the design of this study as GA at delivery did not correlate with storage time (*r* = –0.092; *P* > .41).

### Predictive Modeling of Chronicity of Pregnancy

**Figure 2.**
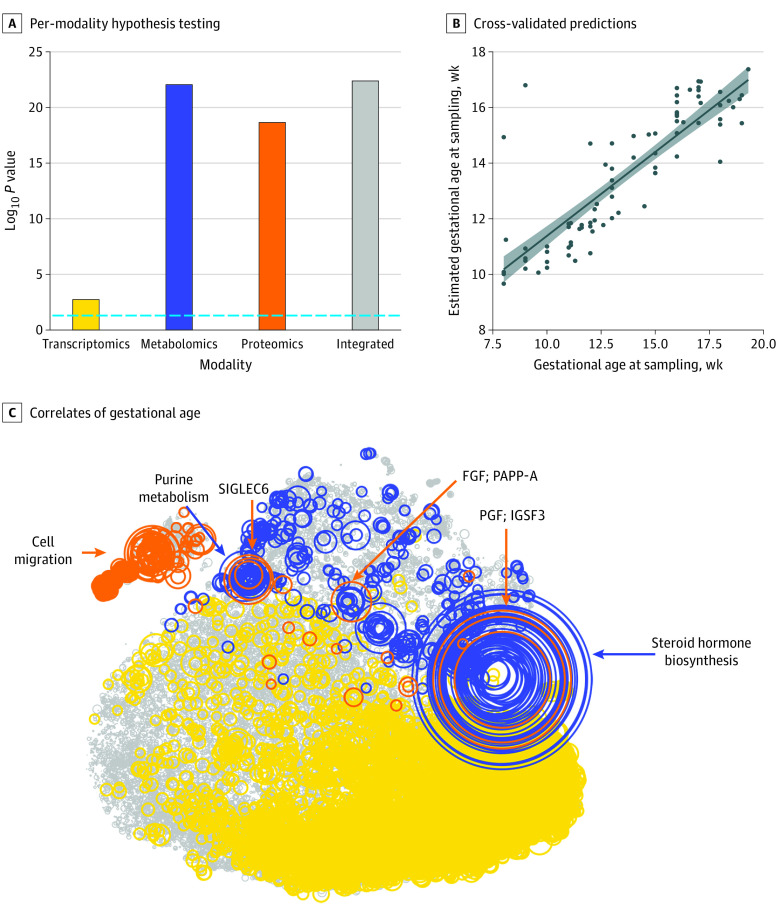
Prediction of Gestational Age (GA) at the Time of Sample Collection A, A cross-validation strategy was used to simultaneously optimize the integrated model and test the performance of the model on previously unseen patients. Models built on all 3 modalities (transcriptomics, metabolomics, and proteomics) and the integrated model were statistically significantly correlated with GA at the time of sample collection (Bonferroni-adjusted Spearman correlation *P* < .05). B, The correlation between GA at the time of sample collection and the estimated values on the blinded samples are shown. The shaded area represents the 95% CI. C, The features correlated with the progression of pregnancy (Spearman correlation *P* < .05) are color-coded according to biological modality. FGF indicates fibroblast growth factor; IGSF3, immunoglobulin superfamily member 3; PAPP-A, pregnancy-associated plasma protein A; PGF, placental growth factor; and SIGLEC6, sialic acid binding Ig-like lectin 6.

We built models to estimate GA at the time of sample collection (as a surrogate for the chronicity of pregnancy) for each patient. A cross-validation strategy was used to simultaneously optimize the integrative model and test the performance of the model on previously unseen patients. Models built on all 3 modalities (transcriptomics, metabolomics, and proteomics) as well as the integrated model were statistically significantly correlated with GA at the time of sample collection (transcriptomics: 1.736089 × 10^−03^; metabolomics: 8.936983 × 10^−23^; proteomics: 2.227379 × 10^−19^; and integrated model: 8.990768 × 10^−22^; Bonferroni-adjusted Spearman correlation *P* < .05) (Figure 2A and B). The features that most correlated with the progression of pregnancy (Spearman correlation *P* < .05) are color-coded in Figure 2C. A cluster of highly correlated metabolomics and proteomics features was identified that included the trophoblast-derived placental growth factor (PGF). Previous studies have demonstrated that PGF plays a substantial role in the pathogenesis of preeclampsia but has not been associated with spontaneous PTB.^28,29^ Pathway analysis^30^ of the metabolites in this module indicated the enrichment of the steroid hormone biosynthesis pathway (Fisher test for pathway enrichment analysis *P* < 1.2 × 10^−12^). The purine metabolism pathway was enriched in an additional module of metabolites (Fisher test for pathway enrichment analysis *P* < 1.7 × 10^−5^). Other proteins that were included in the model and close to this cluster were PAPP-A (pregnancy-associated plasma protein A), MMP-7 (matrix metallopeptidase 7), FGF and FGFBP1 (fibroblast growth factors), and SIGLEC6 (sialic acid binding Ig-like lectin 6), all of which play important roles in placental development.^31,32,33,34^ An additional cluster of proteins associated with cell migration and localization was identified by gene ontology analysis (Protein Analysis Through Evolutionary Relationships overrepresentation *P* < 10 × 10^−7^).

To further highlight the interplay between plasma proteins and urine metabolites, we developed a random forest model to estimate the PGF levels of each patient using only the urine metabolomics data set (eFigure 2 in the Supplement). Overall, this analysis highlighted the potential for biological profiling for estimating GA during pregnancy (a substantial challenge in LMICs) and the use of urine-based metabolite biomarkers as low-cost surrogates for models developed through multiomics analysis.

### Predictive Modeling of PTB

**Figure 3.**
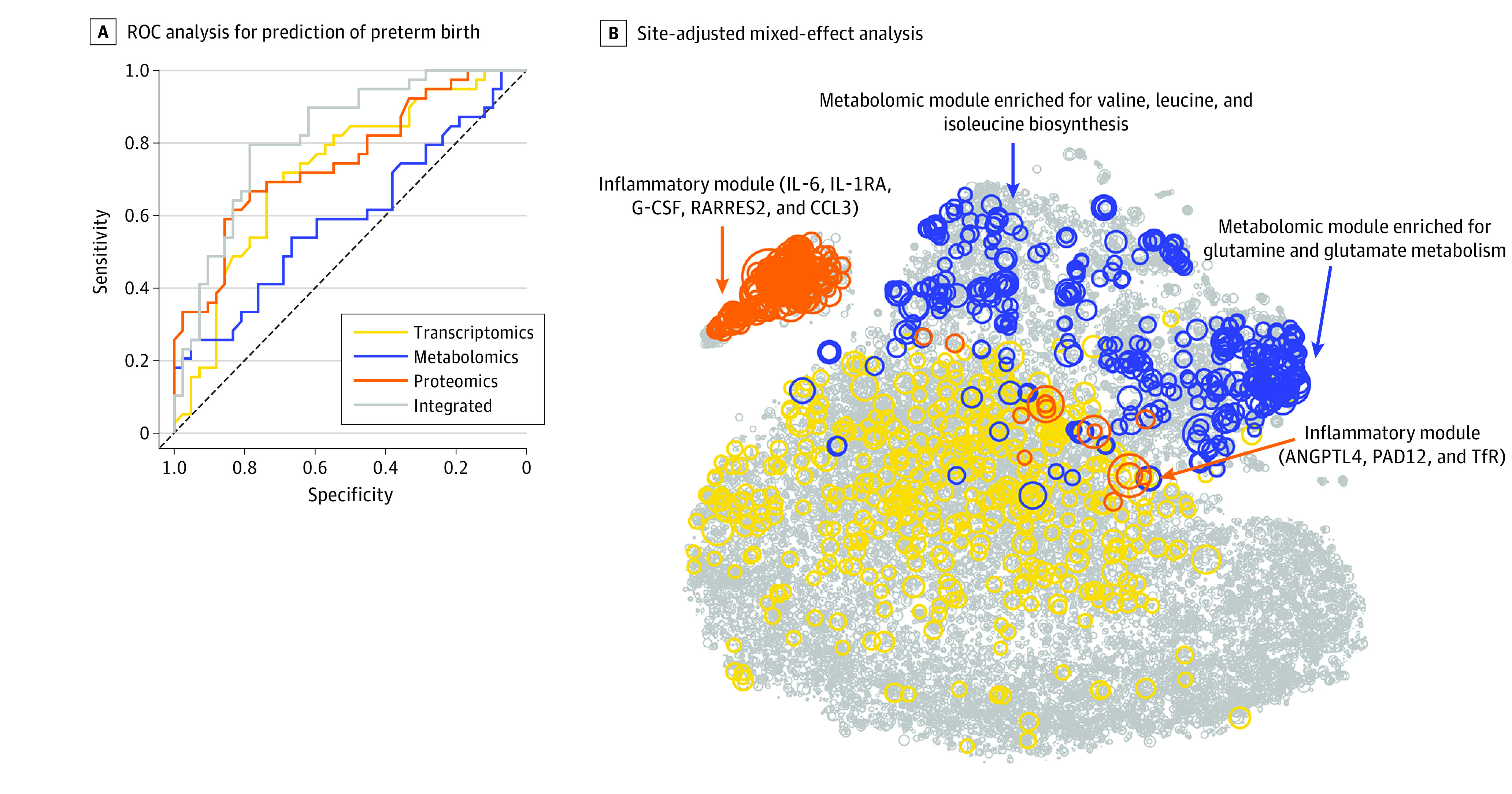
Predictive Modeling of Preterm Birth (PTB) A, This receiver operating characteristic (ROC) curve analysis used each biological modality and the integrated approach. The mean area under the ROC curve and 95% CI for each modality were as follows: transcriptomics (AUROC, 0.73; 95% CI, 0.61-0.83), metabolomics (AUROC, 0.59; 95% CI, 0.47-0.72), proteomics (AUROC, 0.75; 95% CI, 0.64-0.85), and integrated (AUROC, 0.83; 95% CI, 0.72-0.91). B, Circle size is proportional to −log_10_ (Wilcoxon) *P* value for discrimination between term pregnancies and PTBs. Top features included an inflammatory module (which included interleukin 6 [IL-6]; IL-1 receptor antagonist [IL-1RA], a regulatory member of the IL-1 family whose expression is induced IL-1β under inflammatory conditions; granulocyte colony-stimulating factor [G-CSF]; retinoic acid receptor responder protein 2 [RARRES2]; chemokine ligand 3 [CCL3]; angiopoietin-like 4 [ANGPTL4]; protein-arginine deiminase type II [PADI2]; and transferrin receptor [TfR]) and a metabolomic module (which was enriched for glutamine and glutamate metabolism [Fisher test for pathway enrichment analysis *P* < 4.4 × 10^−9^] and valine, leucine, and isoleucine biosynthesis pathways [*P* < 7.3 × 10^−6^]).

For prediction of PTB (GA at delivery <37 weeks), we used a leave-one-out cross-validation procedure to test the models on blinded participants. Before training the model using the entire data set, the feature space was limited to the top features in the cohort that corresponded to the blinded sample based on univariate testing. Overall, the models relied on a subset of all available features. The median number of features used by the models during cross-validation was 36 for transcriptomics, 35 for metabolomics, and 9 for proteomics. To combine predictions from each model, we developed an additional integration layer to produce the final weighted probabilities for statistical testing. The integrated model was more accurate than the model for each independent modality (Figure 3A). The mean area under the receiver operating characteristic curve (AUROC) and 95% CI for each modality were as follows: transcriptomics (AUROC, 0.73; 95% CI, 0.61-0.83), metabolomics (AUROC, 0.59; 95% CI, 0.47-0.72), proteomics (AUROC, 0.75; 95% CI, 0.64-0.85), and integrated (AUROC, 0.83; 95% CI, 0.72-0.91) (Figure 3A). eFigure 3 in the Supplement provides a comparison against other machine learning strategies applied to the same data set (support vector regression AUROC, 0.57; random forest AUROC, 0.66; lasso AUROC, 0.68; Gaussian process AUROC, 0.71; supervised learning cohort-adjusted model AUROC, 0.83; merging AUROC, 0.71; stacked generalization AUROC, 0.76; data integration cohort-adjusted model AUROC, 0.83). In an independent analysis, this same pipeline was used to model participants who were randomly assigned to case and control groups, confirming that the findings presented in Figure 3 did not result from model overfitting (transcriptomics AUROC, 0.54; metabolomics AUROC, 0.50; proteomics AUROC, 0.50; integrated AUROC, 0.50) (eFigure 4 in the Supplement).

Field workers were trained to collect detailed phenotypic and demographic data from the women and their families through scheduled household visits during pregnancy and postpartum. Clinical covariates were manually harmonized across all 5 cohorts. Of all the variables collected, only the weight of the baby and GA at delivery were statistically significantly correlated with the final outcome of the model predicting PTB (Spearman correlation = 0.73). (eFigure 5 and eTable in the Supplement). This finding confirmed that the model was not confounded by the other measured clinical covariates.

Given the statistically significant differences observed across various cohorts, we used mixed-effect models (with each cohort encoded as a random effect) to compare the distribution of each measurement between term pregnancies and PTBs (Figure 3B). Top features were contained within 2 correlated modules: (1) an inflammatory module, which included interleukin 6 (IL-6), IL-1 receptor antagonist (IL-1RA, a regulatory member of the IL-1 family whose expression is induced IL-1β under inflammatory conditions^35,36^), granulocyte colony-stimulating factor (G-CSF), retinoic acid receptor responder 2 (RARRES2), and chemokine ligand 3 (CCL3), and (2) a metabolomic module, which primarily consisted of urine metabolites enriched for glutamine and glutamate metabolism (Fisher test for pathway enrichment analysis *P* < 4.4 × 10^−9^)^30^ and valine, leucine, and isoleucine biosynthesis pathways (*P* < 7.3 × 10^−6^).^37^

The presence of inflammatory mediators among the features correlated with PTB is consistent with finding in previous studies that suggested dysfunctional immune adaptations during pregnancy was central to the pathogenesis of PTB.^38,39^ However, the predictive model also highlighted a set of proteomic features with no known inflammatory properties that were correlated with features from the inflammatory module. These proteins included protein-arginine deiminase type II (PADI2), a peptidylarginine deiminase that is responsible for protein citrullination and implicated in parturition and sensing infections^40,41^; transferrin receptor (TfR), which is implicated in iron transport; angiopoietin-like 4 (ANGPTL4), which regulates glucose homeostasis and lipid metabolism^42^; and RARRES2, an adipokine that is increased in metabolic syndrome and gestational diabetes.^43,44^

To ascertain whether observed correlations between these proteins and the inflammatory module reflected biologically relevant inflammatory properties, we examined the capacity of each of these factors to stimulate human peripheral blood leukocytes using an ex vivo mass cytometry assay.^45^ The activity of major intracellular signaling responses previously^17^ implicated in maternal immune adaptations during pregnancy was assessed at baseline and after a 15-minute stimulation in major innate and adaptive immune cell types (eMethods in the Supplement). As expected, robust and cell-specific signaling responses along the JAK/STAT and MyD88 signaling pathways were observed in classical monocytes (CMC) after stimulation with known proinflammatory cytokines, including IL-6 (mean [SD] pSTAT3 ArcSinh ratio over endogenous signal, 2.64 [0.22]; false discovery rate [FDR]–adjusted vs unstimulated *P* < 1.0 × 10^−6^), G-CSF (mean [SD] pSTAT5 ArcSinh ratio over endogenous signal, 0.42 [0.12]; *P* = .007), and CCL3 (mean [SD] pCREB ArcSinh ratio over endogenous signal, 0.35 [0.09]; *P* < 1.0 × 10^−6^) (eFigures 6 and 7 and the eMethods in the Supplement). Stimulation with PADI2 activated the key elements of the MyD88 pathway, including P38 (mean [SD] ArcSinh ratio over endogenous signal, 0.91 [0.52]; FDR-adjusted vs unstimulated *P* = .007), MK2 (mean [SD] ArcSinh ratio over endogenous signal, 0.38 [0.10]; *P* = .002), and NFkB (mean [SD] ArcSinh ratio over endogenous signal, 0.14 [0.03]; *P* = .009), in monocytes, although little or no signaling responses were observed after stimulation with ANGPTL4, TfR, or RARRES2.

We also tested whether stimulation with the most informative proteomic features of the predictive model of PTB would alter the effector function of circulating immune cells. To this end, we quantified the intracellular expression of select cytokines in circulating immune cells that were stimulated with the target proteins for 4 hours. In addition to the expected cytokine responses after exposure to CCL3, IL-6, and G-CSF, the results show that PADI2 and ANGPTL4 stimulated proinflammatory cytokine production in CMC (mean [SD] frequency of PADI2-stimulated IL-1β + CMC: 18.66 [1.93], FDR-adjusted vs unstimulated *P* < 1.0 × 10^−6^; mean [SD] frequency of PADI2-stimulated IL-6 + CMC: 8.01 [1.47], *P* = 1.0 × 10^−6^; mean [SD] frequency of PADI2-stimulated TNF + CMC: 7.43 [1.44], *P* = 1.0 × 10^−6^) (eFigure 8 and eMethods in the Supplement).

In contrast, stimulation with RARRES2 or TfR elicited little intracellular cytokine responses (mean [SD] frequency of RARRES2-stimulated IL-1β + CMC: 5.63 [0.25], FDR-adjusted vs unstimulated *P* < 1.0 × 10^−6^; mean [SD] frequency of TfR-stimulated IL-1β + CMC: 2.25 [0.66], *P* = .16). These results provide evidence of the potential communication between different biological systems and add new elements to the complex pathogenesis of preterm birth. Furthermore, the results suggest that PADI2, in conjunction with other inflammatory cytokines (such as IL-1β), may exacerbate proinflammatory innate immune responses during PTBs, thereby playing a role in the early onset of labor.

## Discussion

To our knowledge, this study is the first multicohort and multiomics analyses of term and preterm birth conducted in LMICs through use of biorepository samples from relevant geographies in a harmonized fashion. The plasma and urine samples were collected, processed, stored, and shipped to the laboratories under uniform protocols. In this proof-of-concept study, a machine learning approach was implemented for quality control, analysis of the timing of pregnancy, and prediction of PTB. Cohort-specific signatures were observed in all cohorts, and data quality was consistent across all modalities.

The prediction of GA at the time of sample collection was driven by an internally correlated module of placenta-related plasma proteins and urine metabolites. Correlations within this module provided an excellent example of leveraging multiomics data for identification of low-cost surrogates in an accessible biological sample (in this case, urine) for an otherwise complex plasma-based measurement with direct applications in LMICs. Accurate prediction of GA through laboratory testing of blood or urine, if validated in larger and more diverse cohorts, has the potential for widespread implementation in settings in which ultrasonography-based GA dating is not available or is impractical.

Prediction of PTB using a multiomics model adjusted for each cohort resulted in an AUROC of 0.83. The sparse nature of the developed methods indicated the possibility of developing simplified models in a validation cohort for scalable analysis of larger cohorts. Mixed-effect modeling revealed several features of interest. The top-ranked features, including IL-1RA, pointed to promising anti-inflammatory therapy candidates that were under active development.^46^ Although the prediction of GA at the time of sample collection was consistent across all 5 cohorts, models for prediction of PTB required cohort-specific adjustments. This finding is consistent with that in previous publications that indicated that, although the normal chronicity of pregnancy may be shared across populations, pathological pregnancies are likely to be population-specific.^47,48^

Each multiomics data set differed not only across the subcohorts but also in terms of their size and internal complexities. Therefore, we used a 2-step machine learning strategy in which a model was first built on each omics data set and then combined for final predictions. This approach prevented large untargeted data sets from overwhelming small yet carefully targeted assays that could have a similar or even more discriminatory information content. This approach resulted in an increase in predictive power and improved interpretability of the results.

In the present study, the predictive accuracy for PTB was augmented by combining various omics data sets, which was consistent with previous studies suggesting that PTB was a condition manifesting within multiple biological systems.^18,49,50,51,52^ Observed differences between cohorts also highlighted that the causes of PTB may be associated with varying environmental and socioeconomic factors.^53^ From a biological standpoint, examination of individual components of the multiomics model emphasized the role of inflammation in the pathobiological features of PTB. As such, inflammatory cytokines previously shown to be elevated in PTBs, including IL-6 and IL-1RA (often considered as a surrogate marker of IL-1β expression^54^) were among the most informative features of the multiomics model.^55^ These cytokines were integrated within a broader inflammatory module that revealed novel factors associated with preterm labor with previously unsuspected properties (eg, PADI2). In neutrophils, citrullination of histones by PADI2 is an important step in the formation of neutrophil extracellular traps, a defensive immunity tool that allows neutrophils to trap and kill bacteria.^56,57,58,59,60^ Increased soluble PADI2 observed in PTBs may potentially reflect heightened inflammatory responses to a bacterial pathogen, consistent with an infectious cause for PTB. We show that soluble PADI2 can also directly activate proinflammatory signaling pathways and cytokine production in classical monocytes, highlighting a synergistic mechanism that may further enhance the inflammatory state of PTB.

### Strengths and Limitations

This study had several strengths. First, the AMANHI and GAPPS biorepositories used accurate early trimester ultrasonography scans for GA dating. Second, urine and plasma specimens were collected, processed, and transported in a harmonized manner. All samples underwent a single freeze-thaw cycle only at Stanford University before final processing and analysis. Third, the machine learning strategy used was able to detect patterns that were generalizable across cohorts.

This study also had several limitations. First, it used a small sample size compared with the number of measurements (which we accounted for through a rigorous 2-step cross-validation process). Therefore, reproduction of these results in larger and more diverse cohorts remains a major priority for our future efforts. For reproduction of these results to be successful, the validation of a reduced model with increased scalability will be a key step. Second, given the exploratory nature of this study, the cohort was clinically homogeneous (eTable and eFigure 2 in the Supplement), which limits the generalizability of the results to real-world heterogeneous populations. Therefore, a future area of investigation is the direct integration of clinical covariates into the predictive models^61^ to increase the generalizability in data sets with diverse phenotypes.

## Conclusions

This diagnostic/prognostic study found that, in LMICs and high PTB settings, major biological adaptations during pregnancy may follow a generalizable model, but the biological signals that correlate with or are potentially associated with PTB can be detected using robust machine learning algorithms. In addition, this study demonstrated that a multiomics approach has the potential to both improve and help identify low-cost predictive surrogates in accessible biological samples for LMICs. Research to expand this analysis to a larger patient population and to broader cohorts and omics platforms are already under way. The data sets, together with state-of-the-art machine learning partnerships,^62^ will be a key step in developing valuable predictive tests and intervention candidates to tackle the long-term clinical challenge of preventing PTB.

## Notes

Supplement. **eMethods.** **eFigure 1.** Data Quality Assessment **eFigure 2.** Urine Metabolites as a Surrogate for PGF in Plasma **eFigure 3.** Empirical Algorithm Comparison **eFigure 4.** A Lower Bound for the Analysis Pipeline Using a Negative Example **eFigure 5.** Analysis of Clinical Covariates **eTable.** Table of Clinical Covariates Harmonized Across All Cohorts **eFigure 6.** Comprehensive Visualization of Single-Cell-Level Intracellular Signaling in Response to Selected Plasma Proteins **eFigure 7.** Top Proteomics Features Activate Intracellular Signaling Pathways in Peripheral Blood Classical Monocytes **eFigure 8.** Top Proteomics Features Activate Cytokine Production in Peripheral Blood Classical Monocytes **eReferences**
