# Errors in the eTable

**DOI:** 10.1001/jamanetworkopen.2021.0399

**Published:** 2021-02-12

In the Original Investigation titled “Multiomics Characterization of Preterm Birth in Low- and Middle-Income Countries,”^1^ published December 18, 2020, there were errors in the data reported in the History of PTB row of the eTable in the Supplement. The Supplement has been corrected.
