# Supplementary material for: Multiomics Characterization of Preterm Birth in Low- and Middle-Income Countries
Source: JAMA Netw Open. 2020 Dec 18;3(12):e2029655. doi: 10.1001/jamanetworkopen.2020.29655 (PMC7749442; doi:10.1001/jamanetworkopen.2020.29655)
Supplement: Supplement. — eMethods. eFigure 1. Data Quality Assessment eFigure 2. Urine Metabolites as a Surrogate for PGF in Plasma eFigure 3. Empirical Algorithm Comparison eFigure 4. A Lower Bound for the Analysis Pipeline Using a Negative Example eFigure 5. Analysis of Clinical Covariates eTable. Table of Clinical Covariates Harmonized Across All Cohorts eFigure 6. Comprehensive Visualization of Single-Cell-Level Intracellular Signaling in Response to Selected Plasma Proteins eFigure 7. Top Proteomics Features Activate Intracellular Signaling Pathways in Peripheral Blood Classical Monocytes eFigure 8. Top Proteomics Features Activate Cytokine Production in Peripheral Blood Classical Monocytes eReferences [file jamanetwopen-e2029655-s001.pdf]

# Supplementary Online Content

Jehan F, Sazawal S, Baqui AH, et al; Alliance for Maternal and Newborn Health Improvement, Global Alliance to Prevent Prematurity and Stillbirth, and Prematurity Research Center at Stanford University. Multiomics characterization of preterm birth in low- and middle-income countries. *JAMA Netw Open*. 2020;3(12): e2029655. doi: 10.1001/jamanetworkopen.2020.29655


**eMethods.**

**eFigure 1.** Data Quality Assessment

**eFigure 2.** Urine Metabolites as a Surrogate for PGF in Plasma

**eFigure 3.** Empirical Algorithm Comparison

**eFigure 4.** A Lower Bound for the Analysis Pipeline Using a Negative Example

**eFigure 5.** Analysis of Clinical Covariates

**eTable.** Table of Clinical Covariates Harmonized Across All Cohorts

**eFigure 6.** Comprehensive Visualization of Single-Cell-Level Intracellular Signaling in Response to Selected Plasma Proteins

**eFigure 7.** Top Proteomics Features Activate Intracellular Signaling Pathways in Peripheral Blood Classical Monocytes

**eFigure 8.** Top Proteomics Features Activate Cytokine Production in Peripheral Blood Classical Monocytes

**eReferences**


This supplementary material has been provided by the authors to give readers additional information about their work.

©2020 Jehan F et al. *JAMA Network Open*

# eMethods

## 1 Study Design

Inclusion criteria for samples, which consisted of plasma and urine were: collection $\leq$ 20 weeks of GA, and determination of GA by ultrasound ( $<$ 37 weeks' GA for PTBs and $>$ 37 weeks' GA for term births). Medically-indicated preterm deliveries were excluded.

Our study population consisted of 81 pregnant women selected from the following GAPPS- and AMANHI-supported birth cohorts: (1) the GAPPS Preterm and Stillbirth Study in Matlab, Bangladesh (PreSSMat Study, icddr,b, Matlab, Bangladesh), a prospective cohort study designed to assess biological, environmental, and social determinants of adverse pregnancy outcomes; (2) the GAPPS Preventing Preterm Birth Initiative in Zambia (ZAPPS Study, UNC-CH/UTH, Lusaka, Zambia) [8], a prospective cohort study and biorepository designed to characterize the factors associated with PTB and outcomes in Zambia; and (3) the Alliance for Maternal and Neonatal Health Improvement (AMANHI) biorepository study in Bangladesh Sylhet, Pakistan Karachi and Pemba Tanzania. All pregnant women provided written informed consent for participation in the original study, and for future utilization of specimens. For the current studies ethical exemptions were sought from the respective in-country IRBs and regulated under necessary material transfer and data transfer agreements.

At all AMANHI and GAPPS cohorts, trained phlebotomists collected blood samples for centrifugation and aliquoting of serum, plasma, and buffy coat for storage and future analyses. In addition, maternal urine was collected in parallel. With a view to facilitate the future of omics study, special care was taken to ensure sample storage at $-80\,^{\circ}\mathrm{C}$ in each biobank. Unique study identification numbers were assigned to all samples, which were linked to each participant. Outcome assessment was done by birth surveillance through phone calls and household visits [12].

Collection and processing of all sample types was performed following standard operating procedures at all study cohorts [7]. Blood collected in EDTA tubes was cold centrifuged at $3,000$ rpm for 10 mins within 4 hrs. Plasma was separated and stored at $-80\,^{\circ}\mathrm{C}$ until shipment. 1.0 mL of plasma for transcriptome, 0.5 mL of plasma for proteome, and two aliquots of 2 mL each of urine for metabolome analysis were shipped from each biorepository. Samples were shipped on dry ice as a single batch and under continuous temperature monitoring.

©2020 Jehan F et al. *JAMA Network Open*

# 2 Biological Modalities

## 2.1 Transcriptomics

cfRNA was extracted from 1mL of plasma using a Plasma/Serum Circulating and Exosomal RNA Purification mini kit (Norgen, cat510000) following manufacturer's instructions. The residue of DNA was digested using Baseline-ZERO DNase (Lucigen, DB0715K) and then cleaned by RNA Clean and Concentrator-5 kit (Zymo, R1013). RNA was eluted to 12 $\mu$L in the elution buffer.

Eight mL of the eluted RNA was used for sequencing library preparation using SMARTer Stranded Total RNAseq kit v2 -Pico Input Mammalian (Clontech, cat634413) according to the manufacturer's instructions. Short read sequencing was performed using the Illumina NovaSeq S2 2-Lanes ($2 \times 75$ bp) platform to the depth of more than 10 million reads per sample. The sequencing reads were mapped to human reference genome (hg38) using STAR aligner [11]. Duplicates were removed by PICARD [14] and then gene counts were quantified using unique reads with htseq-count [3]. Prior results demonstrated a strong correlation between this assay and RT-qPCR measurements [19].

## 2.2 Metabolomics

Global metabolic profiling of urine samples was performed using a broad spectrum liquid chromatography coupled with mass spectrometry platform (LC-MS). Urine aliquots were prepared and analyzed as previously described [9]. Briefly, urine samples were thawed on ice and centrifuged at $17,000$rcf for 10 minutes. The supernatants were diluted by a factor of four with 75% acetonitrile and 100% water including 13 internal standards (IS) for HILIC- and RPLC-MS experiments, respectively. Samples for HILIC-MS experiments were further centrifuged at $21,000$g for 10 min at 4 °C to precipitate proteins.

Metabolic extracts were analyzed four times using HILIC and RPLC separation in both positive and negative ionization modes. Data were acquired on a Thermo Q Exactive HF mass spectrometer that was equipped with a HESI-II probe and operated in full MS scan mode. MS/MS data were acquired at different fragmentation energies (NCE 25, 35 and 50) on pool samples (QC) consisting of an equimolar mixture of all samples in the study. HILIC experiments were performed using a ZIC-HILIC column 2.1 x 100mm, 3.5$\mu$m, 200Å (Merck Millipore) and mobile phase solvents consisting of 10mM ammonium acetate in 50/50 acetonitrile/water (A) and 10mM ammonium acetate in 95/5 acetonitrile/water (B). RPLC experiments were performed using a Hypersil GOLD column $2.1 \times 150$mm, 1.9$\mu$m, 175Å (Thermo Scientific) and mobile phase solvents consisting of 0.06% acetic acid in water (A) and 0.06% acetic acid in methanol (B).

Data quality was ensured by (1) sample randomization for metabolite extraction and data acquisition, (2) multiple injections of a pool sample to equilibrate the LC-MS system prior to run the sequence (12 and 6 injections for HILIC and RPLC methods, respectively), (3) spike-in labeled IS during sample preparation

©2020 Jehan F et al. *JAMA Network Open*

to control for extraction efficiency and evaluate LC-MS performance, (4) checking mass accuracy, retention time and peak shape of IS in every samples and (5) injection of a pool sample every 10 injections to control for signal deviation with time.

Data from each mode were independently analyzed using Progenesis QI software (v2.3) (Nonlinear Dynamics). Metabolic features from blanks and that did not show sufficient linearity upon dilution in QC samples ($r < 0.6$) were discarded. Only metabolic features present in $> 2/3$ of the samples were kept for further analysis. Inter- and intra-batch variation was corrected using the LOESS (locally estimated scatterplot smoothing Local Regression) normalization method on pool samples injected repetitively along the batches (span = 0.75). Missing values were imputed by drawing from a random distribution of low values in the corresponding sample. Data from each mode were merged to obtain a dataset containing 6,630 putative metabolites. Dilution effect was corrected by using the probabilistic quotient normalization (PQN) [10]. Metabolic features were annotated by matching the experimental accurate mass ($\pm 5$ ppm) to a local database containing $60,000+$ metabolites. This database was created by compiling metabolites from various public databases including HMDB, FoodB and DrugBank [25].

## 2.3 Proteomics

The proteomic analysis was performed by O-link Proteomics (Watertown, MA) with a highly multiplex proteomic platform using proximity extension technology [6]. For this study, eleven panels were used, each measuring 92 different proteins simultaneously in 1$\mu$L of plasma. Each protein was detected by a matched pair of antibodies that were coupled to unique and partially complementary oligonucleotides. When in close proximity, a new and protein-specific DNA reporter sequence was formed by hybridization and extension, which was then amplified and quantified by real-time PCR [20].

Relative amounts of protein were quantified as normalized protein expression (NPX). NPX was derived by subtracting the Ct value of the extension control reaction from the raw Ct-value (threshold cycle) to adjust for technical variations (dCT), then subtracting differences in Ct-values between plates (inter-plate control) from the dCt-value (ddCt-value) to adjust for inter-assay variability, and then subtracting the ddCt-value from a correction factor to adjust for background noise and invert the scale. An increase of 1 NPX corresponded to a doubling of the relative protein concentration ($\log 2$ scale).

Quality control (QC) was performed at the assay and sample level [1]. At the assay level (internal controls) each sample was spiked with two non-human antigens (incubation control), an antibody coupled with a unique pair of DNA tags (extension control), and a double-stranded DNA amplicon (detection control) to monitor the three major procedural steps (immunoreaction, extension, and amplification/detection). At the sample level three controls were added to each plate. A synthetic sample containing 92 antibodies with one pair of unique DNA tags in fixed proximity was added in triplicate to monitor and

compensate for inter-run and inter-plate variations (inter-plate control). A negative control was added in triplicate to monitor for background noise. Finally, a pooled plasma sample was added in duplicate to monitor for intra- and inter-assay variability and determine coefficient of variations. A plate passes QC if the standard deviation of internal controls was less than 0.2 NPX. Individual samples pass QC if values of internal controls deviated by less than 0.3 NPX from the plate median. In this study, the plate passed QC as did 97.7% of the samples. Of all assayed proteins 88.8% were detected in more than 75% of samples. The median intra-assay coefficient of variation was 7%. Prior studies have demonstrated strong associations between this assay and ELISA analysis (*e.g.,* [5, 24, 16, 15].)

## 2.4 Quality Control

Additional control was performed by visualization of all subjects using unsupervised analysis, supplemented by objective and quantitative analysis using a supervised algorithm as described in eFigure 1. While cohort-specific signatures and signatures associated with storage time were observed, overall data quality was consistent across all modalities.

©2020 Jehan F et al. *JAMA Network Open*

## eFigure 1. Data Quality Assessment.

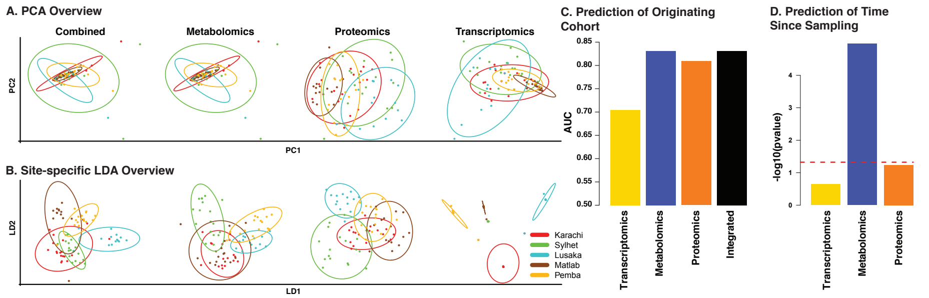


(A) To investigate cohort-specific data signatures, principal component analysis (PCA) was used to create a two-dimensional representation of the entire cohort for each biolog-ical modality as well as all modalities combined. This analysis demonstrated that the largest source of variation in the data was not driven by fundamen-tal differences between the cohorts, underscoring the decreased likelihood that there was bias induced by different sampling or processing protocols. Super-vised linear discriminant analysis (LDA) confirmed the existence of more subtle cohort-specific signatures that were not significant enough to be visualized in an unsupervised PCA. (C) The presence of cohort-specific signatures was con-firmed using random forest analysis (subject to cross-validation for prediction of the sampling site of previously unseen patients exclusively based on each bi-ological modality. Overall, this confirmed the presence of consistent yet limited cohort-specific variations in the datasets. (D) The impact of sample storage time was quantified with random forest analysis subjected to cross-validation in which the number of days between sample collection and laboratory anal-yses was used as a continuous prediction target. The random forest results on previously unseen patients were statistically significant only in the case of the urine metabolomics dataset, indicating the potential for sample degradation over time. However, this did not confound the design of this study as gestational age (GA) at delivery did not correlate with storage time ($p > 0.41$, $r = -0.092$).

©2020 Jehan F et al. *JAMA Network Open*

eFigure 2. Urine Metabolites as a Surrogate for PGF in Plasma

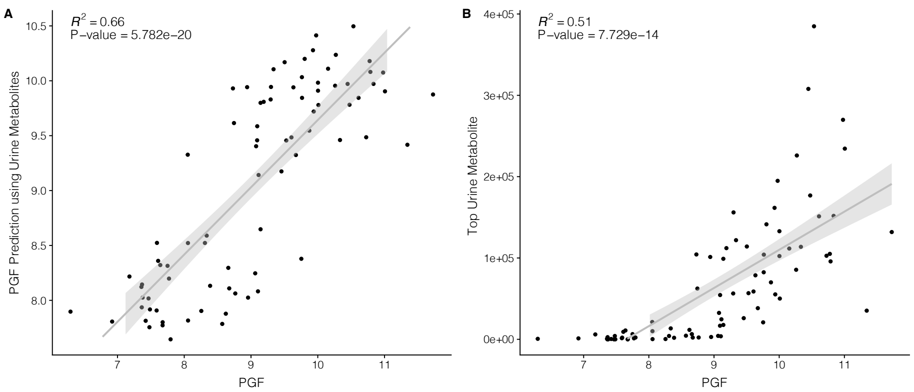


To further highlight the interplay between plasma proteins and urine metabolites, we developed a random forest model to estimate PGF levels of each patient using only the urine metabolomics dataset. (A) A multivariate model produced a strong correlation of plasma PGF in blinded samples; (B) The top feature of the model was strongly correlated with PGF in an independent univariate analysis. Taken together, this analysis highlighted the potential for biological profiling for estimation of gestational age during pregnancy (a significant challenge in LMIC settings) as well as the utility of urine-based metabolite biomarkers as low-cost surrogates for models developed using multiomics analysis.

©2020 Jehan F et al. *JAMA Network Open*

# 3 Computational Analysis

## 3.1 Multivariate Predictive Modeling

Previous bioinformatics work detailing multiomics data integration fall within two major categories: multi-staged, in which measurements of the same biological factors (e.g., genes) are available for alignment of the feature space [22]; and meta-dimensional, in which direct connections between the measured features are not available a priori [21], [13]. Given the diversity of the biological modalities that must be integrated in this study and the lack of preexisting biological connections between all measured factors, a meta-dimensional approach was designed in which each modality is first analyzed independently, and then combined with a higher level integration layer to increase predictive power. Multivariate predictive modeling was performed using a random forest algorithm as implemented in [18] using default parametrization. A comparison against other machine learning algorithms using a similar cross-validation strategy is presented in eFigure 3A. To ensure the generalizability of the models, a Leave-One-Out Cross-Validation (LOOCV) strategy was used to test the predictions on previously unseen patients. In this setting, a model was trained on all available patients except for one. The model was then tested on the blinded subjects. This process was repeated for all subjects until a blinded prediction was calculated for all patients. Final results were reported using these blinded predictions. Cross-validation folds were synchronized be-tween the models built on individual omics datasets and the integrated model to leave out the same data points at all levels of the analysis. Importantly, this guaranteed that not only the aggregate model, but also its input features (*i.e.* the final predictions from each dataset) were blinded to the same subject during cross-validation.

For the prediction of PTB, to account for cohort-specific signatures, we implemented an additional variable-filtering step to reduce the overall search space. Specifically, for a data matrix $X^O$ of $J$ features from $O$ omics platforms

$IJ$

corresponding to cohort $I$, we train a RF model $\mathbf{\Xi}_I = \mathrm{RF}^{\mathrm{Train}}(\mathbf{X}^O_{-iJ_I})$ where $\mathbf{X}^O_{-iJ_I} = \{i' \neq i \wedge \mathbf{X_{i'J_I}} \in \mathbf{X}_{IJ_I}\}$ denotes the removal of patient $i$ from the analysis for cross-validation and $J_I$ is the set of features that are selected by statistical testing between term and preterm cases on $\mathbf{X}_{-iJ_I}$. After training, the blinded prediction $p^O_{I_i} = \mathrm{RF}^{\mathrm{Predict}}(\mathbf{\Xi}_I, \mathbf{X}^O_{I_iJ_I})$ can be calculated for each omics dataset and combined into the final prediction vector $\hat{y}_I = \sum_{k=1}^{3} \omega_I^{o_k} p_I^{o_k} / \sum_{k=1}^{3} \omega_I^{o_k}$ where $\omega_I^{o_k}$ is the classification performance of omics dataset $k$, on cohort $I$, which was calculated using an internal nested cross-validation layer. A comparison against other integration strategies (simple merging of all datasets and stacked generalization) using a similar cross-validation strategy is presented in eFigure 3B.

To calculate a lower bound for the analysis pipeline (to confirm that the strong results are not due to a coding problem that results in information leakage in the cross-validation scheme), a negative example using random data was used. The poor performance of the model on random data (eFigure 4)

confirms that that the strong performance in the real dataset is not due to model overfitting.

eFigure 3. Empirical Algorithm Comparison

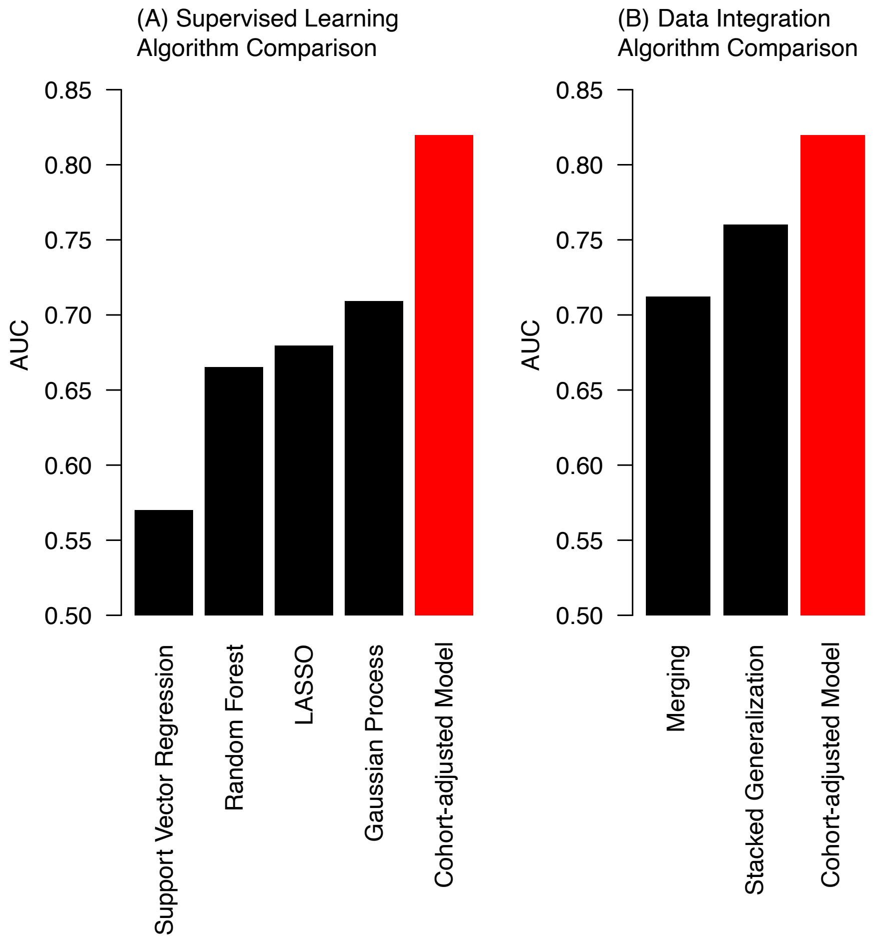


(A) Comparison of supervised learning algorithms for prediction of PTB. All algorithms were evaluated using the same cross-validation strategy described in the meth-ods section. (B) Comparison of multiomics data integration strategies using Random Forest including Merging (where all features are simply merged into a single feature matrix for supervised analysis), Stack generalization (where each dataset is analyzed separately followed by a higher level model combining the results), and the cohort-adjusted pipeline implemented in this article.

eFigure 4. A Lower Bound for the Analysis Pipeline using a Negative Example

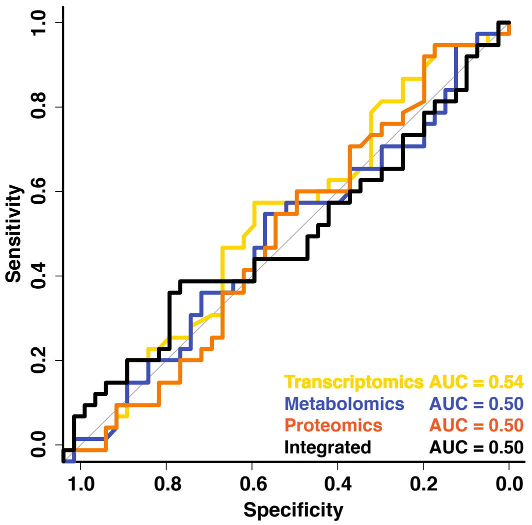


To validate the computational pipeline, data from all patients were randomly assigned to either a case or a control group. The three biological modalities were used to predict these random labels. The pipeline used in Figure 2 was not able to predict the randomly created labels subject to cross-validation. This indicates that the strong performance of the algorithm was not due to model overfitting.

## 3.2 Mixed-Effect Modeling

To account for cohort-specific variations, we employed a linear mixed effect model with cohort encoded as a random effect. Particularly $Y_{is} = \beta_0 + S_{0s} + \beta_1 X_i + e_{is}$, where $e_{is} \sim \mathcal{N}(0, \sigma^2)$ and $Y_{is}$ is a binary vector indicating PTB for patient $i$ in cohort $s$, with a fixed-term intercept $\beta_0$, fixed-term slope $\beta_1$, fixed-term predictor variable $X_i$, random-effect intercept $S_{0s}$ for cohort $s$, and observation-level error $e_{is}$ for patient $i$ in cohort $s$ with variance $\sigma^2$.

## 3.3 Multiomics Visualization

The features from the three omics data were visualized using a dimension reduction strategy designed to balance the size and modularity of each dataset. The top 10 PCs of each omics dataset were used as a 30-dimensional latent space. The correlation matrix between each measurement and this latent space was visualized using the tSNE dimension reduction algorithm [18]. This ensures equal contributions to the visualization layout by all datasets.

©2020 Jehan F et al. *JAMA Network Open*

# 4 Clinical Covariates

Field workers were trained to collect detailed phenotypic and demographic data from the women and their families through scheduled household visits during pregnancy and post-partum. Clinical covariates were manually harmonized across all five cohorts. Of all variables collected, only the weight of the baby and gestational age at delivery were significantly correlated with the final outcome of the model predicting PTB (Supplemental Table S1 and Supplemental Figure S5). This confirmed that the model was not confounded by the other measured clinical covariates.

eFigure 5. Analysis of Clinical Covariates

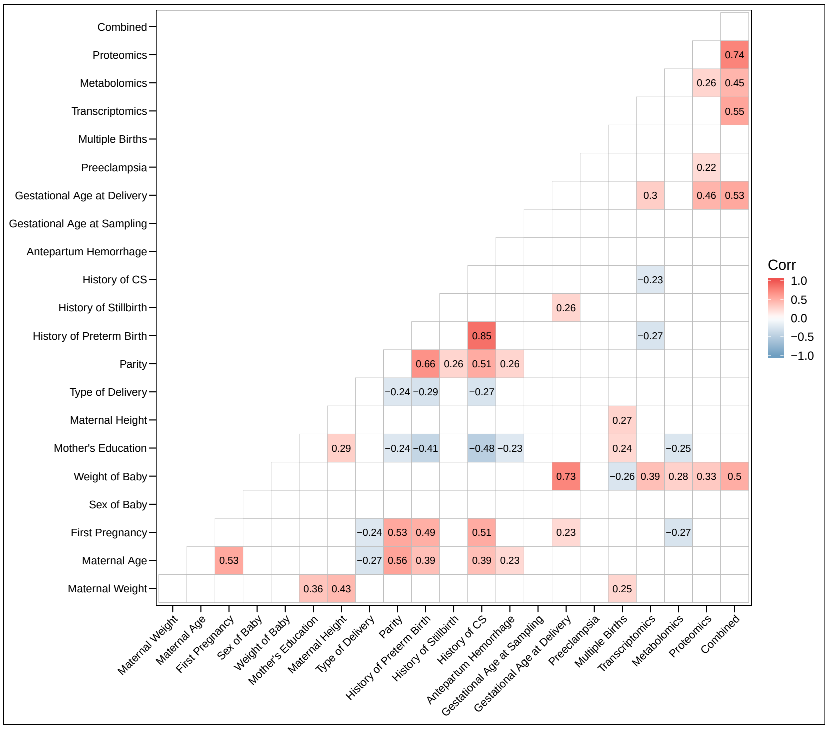


Spearman correlation between the available covariates and the final prediction by each model is visualized. The final combined model was only correlated with gesta-tional age at delivery as well as the weight of the baby.

©2020 Jehan F et al. *JAMA Network Open*

eTable. Table of clinical covariates harmonized across all cohorts.

| | TERM | PRETERM | ALL |
|---|---|---|---|
| n | 42(51.9%) | 39(48.1%) | 81(100%) |
| GA (wks) | $39.8 \pm 0.8$ | $31.6 \pm 2.8$ | $35.9 \pm 4.6$ |
| | (39.0–41.4) | (24.0–36.6) | (24.0–41.4) |
| GA @ Sampling (wks) | $13.7 \pm 3.4$ | $13.6 \pm 3.1$ | $13.6 \pm 3.2$ |
| | (8.0–19.3) | (8.0–18.6) | (8.0–19.3) |
| Parity | $1.8 \pm 1.5$ | $1.6 \pm 1.9$ | $1.7 \pm 1.7$ |
| | (0–5) | (0–8) | (0–8) |
| Maternal age (yr) | $25.0 \pm 4.7$ | $24.5 \pm 6.0$ | $24.8 \pm 5.3$ |
| | (17–39) | (16–39) | (16–39) |
| Maternal weight (kg) | $52.9 \pm 9.8$ | $52.6 \pm 10.9$ | $52.7 \pm 10.3$ |
| | (41.0–82.0) | (29.9–76.6) | (29.9–82.0) |
| Maternal Height (cm) | $154.7 \pm 5.6$ | $153.2 \pm 8.3$ | $154.0 \pm 7.0$ |
| | (141.4–170.0) | (137.0–173.0) | (137.0–173.0) |
| Smoker | 0/35(0.0%) | 0/30(0.0%) | 0/65(0.0%) |
| Prev Stillbirth | 6/42(14.3%) | 2/39(5.1%) | 8/81(9.9%) |
| History of PTB | 27/42(64.3%) | 24/39(61.5%) | 51/81(63.0%) |
| History of Eclampsia | 0/42(0.0%) | 0/33(0.0%) | 0/75(0.0%) |
| History of Pre-Eclampsia | 1/42(2.4%) | 2/39(5.1%) | 3/81(3.7%) |
| Gestational Hypertension | 1/42(2.4%) | 5/33(15.2%) | 6/75(8.0%) |
| | (1.0–4.0) | (1.0–11.3) | (1.0–11.3) |
| Males | 19/42(45.2%) | 18/38(47.4%) | 37/80(46.3%) |
| Females | 23/42(54.8%) | 20/38(52.6%) | 43/80(53.7%) |
| Maternal Disease | | | |
| Thyroid | 0/28(0.0%) | 1/23(4.3%) | 1/51(2.0%) |
| Cancer | 0/37(0.0%) | 0/29(0.0%) | 0/66(0.0%) |
| Epilepsy | 0/28(0.0%) | 0/24(0.0%) | 0/52(0.0%) |
| Mental Illness | 0/28(0.0%) | 0/23(0.0%) | 0/51(0.0%) |
| Malaria | 1/28(3.6%) | 0/23(0.0%) | 1/51(2.0%) |
| Hepatitis B | 1/28(3.6%) | 0/23(0.0%) | 1/51(2.0%) |
| Hepatitis C | 1/28(3.6%) | 0/23(0.0%) | 1/51(2.0%) |
| Urinary Tract Infection | 0/26(0.0%) | 1/20(5.0%) | 1/46(2.2%) |
| Renal | 0/28(0.0%) | 0/22(0.0%) | 0/50(0.0%) |
| Chronic Hypertension | 1/38(2.6%) | 1/29(3.4%) | 2/67(3.0%) |
| Cardiac | 0/37(0.0%) | 1/30(3.3%) | 1/67(1.5%) |
| Diabetes | 0/37(0.0%) | 0/29(0.0%) | 0/66(0.0%) |
| HIV | 0/28(0.0%) | 0/23(0.0%) | 0/51(0.0%) |
| Tuberculosis | 0/37(0.0%) | 1/29(3.4%) | 1/66(1.5%) |
| Other | 2/28(7.1%) | 1/24(4.2%) | 3/52(5.8%) |

©2020 Jehan F et al. *JAMA Network Open*

## 5 Ex Vivo Whole-blood Immuno-assay

The presence of inflammatory mediators among the features most correlated with PTB is consistent with previous studies suggesting that dysfunctional immune adaptations during pregnancy is central to the pathogenesis of PTB. However, the predictive model also highlighted a set of proteomic features with no known inflammatory properties, that were highly correlated with features from the inflammatory module. These proteins included, protein-arginine deiminase type II (PADI2), a peptidylarginine deiminase responsible for protein citrullination and implicated in parturition and sensing infections [17, 4]; transferrin receptor (TfR) which is implicated in iron transport; angiopoietin-like 4 (ANGPTL4) which regulates glucose homeostasis and lipid metabolism (48); and RARRES2, an adipokine increased in metabolic syndrome and gestational diabetes [23, 26]. To determine whether observed correlations between these proteins and the inflammatory module reflected biologically-relevant inflammatory properties, we examined the capacity of each of these factors to stimulate human peripheral blood leukocytes using an ex-vivo mass cytometry assay.

Mass cytometry, an advanced flow cytometry technique, is capable of measuring up to 50 markers in hundreds of thousands of single cells, resulting in detailed functional profiling of all major immune cell types. Using this assay, the activity of major intracellular signaling responses previously implicated in maternal immune adaptations during pregnancy (including pSTAT1, pSTAT3, pSTAT5, pSTAT6, pP38, pMK2, pERK, prpS6, pNFkB, and total IkB) were assessed at baseline and after a 15 minutes stimulation in all major innate and adaptive immune cell-types. Whole blood was collected from healthy, non-pregnant volunteers and stimulated for 15 minutes at 37°C with lipopolysaccharide (1 ug/mL, InvivoGen) and interferon alpha (100 ng/mL, PBL Assay Science),Transferrin Receptor (1 ug/mL, R&D Systems), ANGPTL4 (1 ug/mL, R&D Systems), PADI2 (5 ug/mL, Abnova), RARRES2 (1 ug/mL, R&D Systems), CCL3(1 ug/mL, Invitrogen), G-CSF (100 ng/mL, R&D Systems), and IL-6 (100 ng/mL, R&D Systems) or left unstimulated.

To investigate functional responses to stimulation, cytokine production (IFN$\gamma$, IL-1$\beta$, IL-2, IL-6, IL-4, IL-17A, TNF$\alpha$) and proliferation (Ki67) was assessed in circulating immune cells. Whole blood was collected from healthy, non-pregnant volunteers and stimulated for 4h at 37°C with lipopolysaccharide (1 ug/mL, InvivoGen) and interferon alpha (100 ng/mL, PBL Assay Science), or Transferrin Receptor (1 ug/mL, RD Systems), ANGPTL4 (2.5 ug/mL, RD Systems), PADI2 (2.5 ug/mL, Abnova), RARRES2 (1 ug/mL, RD Systems), CCL3 (1 ug/mL, Invitrogen), G-CSF (100 ng/mL, RD Systems), and IL-6 (100 ng/mL, RD Systems) or left unstimulated, in the presence of Golgi stop and plug (monensin and brefeldin, 1x, BD).

Samples were processed using a standardized protocol for fixation (Smart Tube Inc), barcoding, and staining with antibodies for mass cytometry by time of flight analysis (CyTOF), as described previously [2].

©2020 Jehan F et al. *JAMA Network Open*

eFigure 6. Comprehensive Visualization of Single-cell-level Intracellular Signaling in Response to Selected Plasma Proteins

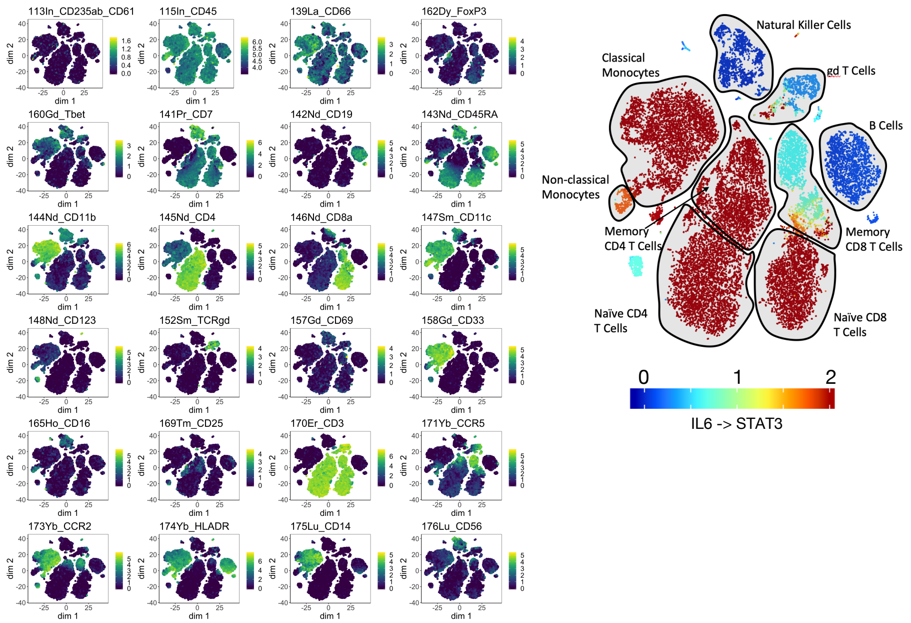


24,000 cells pooled from all samples were visualized using the tSNE algorithm to provide an overview of the expression profiles of all markers (left). Major cell types are annotated in the right panel. Signaling response of pSTAT3 to stimulation by IL6 is used as an example to demonstrate the celltype-specificity of the signaling response.

©2020 Jehan F et al. *JAMA Network Open*

eFigure 7. Top Proteomics Features Activate Intracel-lular Signaling pathways in Peripheral Blood Classical Monocytes

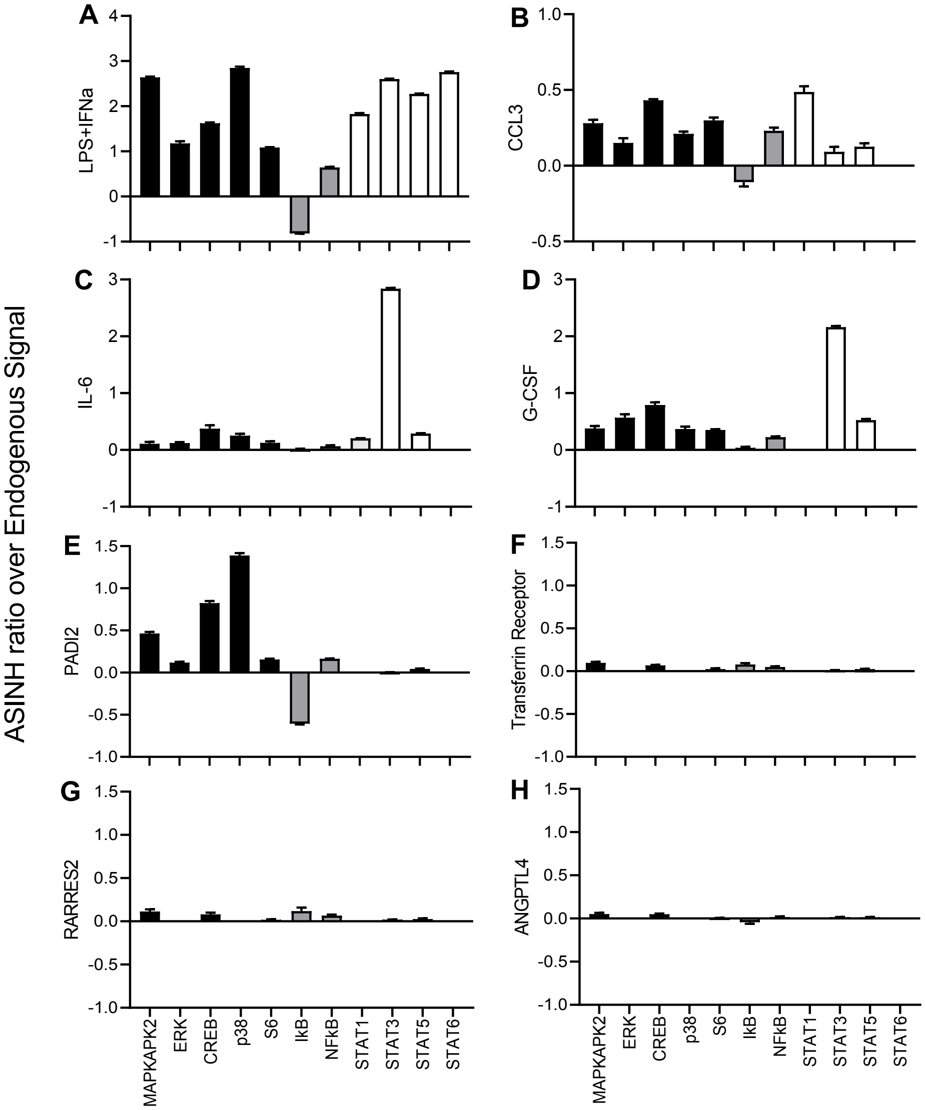


(A) Lipopolysaccharide (LPS) and interferon alpha (IFN-a) together activate MAP Kinase, MyD88, and JAK-STAT signaling pathways (positive control), as shown by phosphorylation of canonical signaling proteins in classical monocytes (cMC, CD14+CD16-) after 15min of incubation. (B) CCL3 stimulation induces a response in MAP Kinase, MyD88, and JAK-STAT signaling pathways. Stimulation with (C) IL-6 and (D) G-CSF induces phosphorylation of STAT3. (E) PADI2 activates key elements of the MyD88 pathway. Stimulation with (F) TfR, (G) RARRES2, and (H) ANGPTL4 did not result in enhanced intracellular signaling activities. Results are representative of three independent experiments each with similar result. Bars represent Mean ± Standard Error.

eFigure 8. Top Proteomics Features Activate Cytokine Production in Peripheral Blood Classical Monocytes

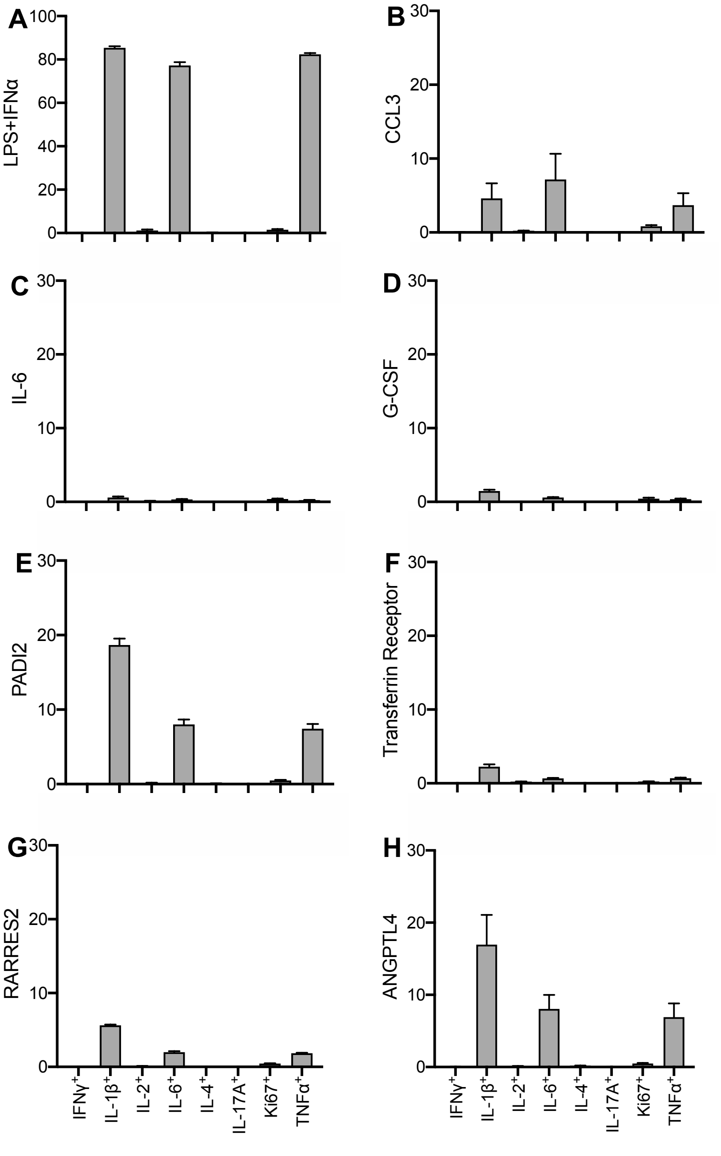


(A) Lipopolysac-charide (LPS) and interferon alpha (IFN-a) together stimulate pro-inflammatory cytokine production (positive control), as shown by the frequency of classical monocytes (cMC, CD14+CD16-) positive for IL-1$\beta$, IL-6, and TNF$\alpha$ after 4h of incubation. (B) CCL3 stimulation induces a similar, albeit lower cytokine response, while cMC are less responsive to stimulation with (C) IL-6 and (D) G-CSF. (E) PADI2 and (H) ANGPTL4 activate production of pro-inflammatory cytokines IL-1$\beta$, IL-6, and TNF$\alpha$. Stimulation with (F) TfR, and (G) RAR-RES2, does result in relatively lower cytokine production in cMC. Results are representative of three independent experiments each with similar result. Bars
representsta Mean $\pm$ Standard Error.

©2020 Jehan F et al. *JAMA Network Open*

# 6 Data Repositories and Source Code

The measured features from all three omics datasets, the algorithms and source codes for reproduction of the results, as well as an interactive website capable of visualizing the entire dataset, the feature evaluation scores for PTB and GA at sampling, and pathway enrichment analysis is available at:
https://nalab.stanford.edu/multiomicsmulticohortpreterm/

# References

[1] *Uppsala, Sweden: Olink Proteomics*, 2019, May 2018.

[2] Nima Aghaeepour, Edward A Ganio, David Mcilwain, Amy S Tsai, Martha Tingle, Sofie Van Gassen, Dyani K Gaudilliere, Quentin Baca, Leslie McNeil, Robin Okada, et al. An immune clock of human pregnancy. *Science immunology*, 2(15), 2017.

[3] Simon Anders, Paul Theodor Pyl, and Wolfgang Huber. Htseq — a python framework to work with high-throughput sequencing data. *Bioinformatics*, 31(2):166–169, Jan 2015.

[4] Tomoji Arai, Masashi Kusubata, Tetsuya Kohsaka, Masakazu Shiraiwa, Kiyoshi Sugawara, and Hidenari Takahara. Mouse uterus peptidylarginine deiminase is expressed in decidual cells during pregnancy. *Journal of cellular biochemistry*, 58(3):269–278, 1995.

[5] Prabhu S Arunachalam, Florian Wimmers, Chris Ka Pun Mok, Ranawaka APM Perera, Madeleine Scott, Thomas Hagan, Natalia Sigal, Yupeng Feng, Laurel Bristow, Owen Tak-Yin Tsang, et al. Systems biological assessment of immunity to mild versus severe covid-19 infection in humans. *Science*, 369(6508):1210–1220, 2020.

[6] Erika Assarsson, Martin Lundberg, Göran Holmquist, Johan Björkesten, Stine Bucht Thorsen, Daniel Ekman, Anna Eriksson, Emma Rennel Dickens, Sandra Ohlsson, Gabriella Edfeldt, et al. Homogenous 96-plex pea immunoassay exhibiting high sensitivity, specificity, and excellent scalability. *PloS one*, 9(4), 2014.

[7] Mandar Bawadekar, Daeun Shim, Chad J Johnson, Thomas F Warner, Ryan Rebernick, Dres Damgaard, Claus H Nielsen, Ger J M Pruijn, Jeniel E Nett, and Miriam A Shelef. Peptidylarginine deiminase 2 is required for tumor necrosis factor alpha-induced citrullination and arthritis, but not neutrophil extracellular trap formation. *Journal of Autoimmunity*, 80:39–47, Jun 2017.

[8] MC Castillo, NM Fuseini, K Rittenhouse, JT Price, BL Freeman, H Mwape, J Winston, N Sindano, C Baruch-Gravett, BH Chi, MP Kasaro, JA Litch, JSA Stringer, and B Vwalika. The zambian preterm birth prevention study (zapps): Cohort characteristics at enrollment. *Gates Open Research*, 2(25), 2019.

[9] Kévin Contrepois, Lihua Jiang, and Michael Snyder. Optimized analytical procedures for the untargeted metabolomic profiling of human urine and plasma by combining hydrophilic interaction (hilic) and reverse-phase liquid chromatography (rplc)–mass spectrometry. *Molecular & Cellular Proteomics*, 14(6):1684–1695, 2015.

©2020 Jehan F et al. *JAMA Network Open*

[10] Frank Dieterle, Alfred Ross, Götz Schlotterbeck, and Hans Senn. Probabilistic quotient normalization as robust method to account for dilution of complex biological mixtures. application in 1h nmr metabonomics. *Analytical chemistry*, 78(13):4281–4290, 2006.

[11] Alexander Dobin, Carrie A Davis, Felix Schlesinger, Jorg Drenkow, Chris Zaleski, Sonali Jha, Philippe Batut, Mark Chaisson, and Thomas R Gingeras. Star: ultrafast universal rna-seq aligner. *Bioinformatics*, 29:15–21, Jan 2013.

[12] AMANHI (Alliance for Maternal, Newborn Health Improvement) Bio–banking Study group), Abdullah H Baqui, Rasheda Khanam, Mohammad Sayedur Rahman, Aziz Ahmed, Hasna Hena Rahman, Mamun Ibne Moin, Salahuddin Ahmed, Fyezah Jehan, Imran Nisar, and et al. Understanding biological mechanisms underlying adverse birth outcomes in developing countries: protocol for a prospective cohort (amanhi bio-banking) study. *Journal of global health*, 7(2):021202, Dec 2017.

[13] Francine E Garrett-Bakelman, Manjula Darshi, Stefan J Green, Ruben C Gur, Ling Lin, Brandon R Macias, Miles J McKenna, Cem Meydan, Tejaswini Mishra, Jad Nasrini, et al. The nasa twins study: A multidimensional analysis of a year-long human spaceflight. *Science*, 364(6436):eaau8650, 2019.

[14] Broad Institute. Picard tools, 2019.

[15] Brynja Jónsdóttir, Marie Ziebell Severinsen, Fredrik von Wowern, Carmen San Miguel, Jens P Goetze, and Olle Melander. St2 predicts mortality in patients with acute hypercapnic respiratory failure treated with noninvasive positive pressure ventilation. *International journal of chronic obstructive pulmonary disease*, 14:2385, 2019.

[16] Anna Tancin Lambert, Xiang Y Kong, Barbara Ratajczak-Tretel, Dan Atar, David Russell, Mona Skjelland, Vigdis Bjerkeli, Karolina Skagen, Matthieu Coq, Eric Schordan, et al. Biomarkers associated with atrial fibrillation in patients with ischemic stroke: A pilot study from the nor-fib study. *Cerebrovascular Diseases Extra*, 10(1):11–20, 2020.

[17] Sigrun Lange. Peptidylarginine deiminases as drug targets in neonatal hypoxic–ischemic encephalopathy. *Frontiers in neurology*, 7:22, 2016.

[18] Andy Liaw, Matthew Wiener, et al. Classification and regression by randomforest. *R news*, 2(3):18–22, 2002.

[19] Thuy TM Ngo, Mira N Moufarrej, Marie-Louise H Rasmussen, Joan Camunas-Soler, Wenying Pan, Jennifer Okamoto, Norma F Neff, Keli Liu, Ronald J Wong, Katheryne Downes, et al. Noninvasive blood tests for fetal development predict gestational age and preterm delivery. *Science*, 360(6393):1133–1136, 2018.

©2020 Jehan F et al. *JAMA Network Open*

[20] Technology overview. Pea: An enabling technology for high- multiplex protein biomarker discovery. *Olink Proteomics*, Dec 2017.

[21] Brian D Piening, Wenyu Zhou, Kévin Contrepois, Hannes Röst, Gucci Jijuan Gu Urban, Tejaswini Mishra, Blake M Hanson, Eddy J Bautista, Shana Leopold, Christine Y Yeh, et al. Integrative personal omics profiles during periods of weight gain and loss. *Cell systems*, 6(2):157–170, 2018.

[22] Eric E Schadt, John Lamb, Xia Yang, Jun Zhu, Steve Edwards, Debraj Guhathakurta, Solveig K Sieberts, Stephanie Monks, Marc Reitman, Chunsheng Zhang, and et al. An integrative genomics approach to infer causal associations between gene expression and disease. *Nature Genetics*, 37(7):710–717, Jul 2005.

[23] Mireille NM van Poppel, Willibald Zeck, Daniela Ulrich, Eva-Christina Schest, Birgit Hirschmugl, Uwe Lang, Christian Wadsack, and Gernot Desoye. Cord blood chemerin: differential effects of gestational diabetes mellitus and maternal obesity. *Clinical endocrinology*, 80(1):65–72, 2014.

[24] Seraina von Moos, Stephan Segerer, Andrew Davenport, Malha Sadoune, Kerem Gerritsen, Julien Pottecher, Frank Ruschitzka, Alexandre Mebazaa, Mattia Arrigo, and Pietro E Cippà. Vascular endothelial growth factor d is a biomarker of fluid overload in haemodialysis patients. *Nephrology Dialysis Transplantation*, 2020.

[25] David S Wishart, Yannick Djoumbou Feunang, Ana Marcu, An Chi Guo, Kevin Liang, Rosa Vázquez-Fresno, Tanvir Sajed, Daniel Johnson, Carin Li, Naama Karu, et al. Hmdb 4.0: the human metabolome database for 2018. *Nucleic acids research*, 46(D1):D608–D617, 2018.

[26] Zhongwei Zhou, Hongmei Chen, Huixiang Ju, and Mingzhong Sun. Circulating chemerin levels and gestational diabetes mellitus: A systematic review and meta-analysis. *Lipids in health and disease*, 17(1):169, 2018.

©2020 Jehan F et al. *JAMA Network Open*
